# Structural and Genetic Identification of the O-Antigen from an *Escherichia coli* Isolate, SD2019180, Representing a Novel Serogroup

**DOI:** 10.3390/ijms242015040

**Published:** 2023-10-10

**Authors:** Jing Wang, Chunjun Qin, Yujuan Xu, Jian Yin, Jing Hu, Xi Guo

**Affiliations:** 1 TEDA Institute of Biological Sciences and Biotechnology, Nankai University, 23 Hongda Street, TEDA, Tianjin 300457, China; 2 Key Laboratory of Carbohydrate Chemistry and Biotechnology, Ministry of Education, School of Biotechnology, Jiangnan University, Lihu Ave. 1800, Wuxi 214122, China; 3 Wuxi School of Medicine, Jiangnan University, Lihu Ave. 1800, Wuxi 214122, China

**Keywords:** *Escherichia coli*, serogroup, O-antigen, structure, O-antigen gene cluster

## Abstract

The O-antigen is one of the outermost surface components of Gram-negative bacteria. Its large structural variation provides the molecular basis for bacterial serological diversity. Here, we established the structure of the O-antigen from an *Escherichia coli* strain, SD2019180, which appeared to be completely different from the known *E. coli* serogroups. The O-antigen tetrasaccharide biological repeating unit was identified as → 2)-[β-d-Glc*p*A-(1 → 4)]-[α-d-Gal*p*-(1 → 3)]-α-l-Fuc*p*-(1 → 3)-α-d-Glc*p*NAc-(1 →. Furthermore, we analyzed the O-antigen gene cluster of SD2019180 and confirmed its role in O-antigen synthesis by using deletion and complementation experiments. Our findings indicate that SD2019180 is a novel serogroup of *Escherichia coli*.

## 1. Introduction

Lipopolysaccharide (LPS) is a component of the outer membrane of Gram-negative bacteria and contributes to various biological functions [1]. It typically consists of a hydrophobic glycolipid, namely, lipid A, a non-repeating core oligosaccharide, and a polysaccharide containing a number of oligosaccharide repeating units (O-units), each with two to eight monosaccharide residues, known as the O-antigen [2]. Because of the great diversity in sugar composition and related linkages within them, the O-antigen is one of the most variable cell constituents [3].

*Escherichia coli* (*E. coli*) is a predominant facultative anaerobe of the human colonic flora, which includes mainly commensal but also pathogenic bacterial strains [4]. The O-antigen is an important virulence factor that influences the survival, invasion, and virulence of *E. coli*; several O-antigen forms are disproportionately represented in pathogenic clones [5,6,7]. The variability in O-antigens provides the basis for the antigenic schemes of many Gram-negative bacteria [8]. The genes coding for the O-antigen are normally clustered at a specific chromosomal locus, namely, the O-antigen gene cluster (O-AGC), which usually maps between the *galF* and *gnd* genes in *E. coli*. Typically, O-AGC comprises three classes of genes, i.e., genes involved in the synthesis of nucleotide sugar precursors, genes encoding glycosyltransferase, and genes responsible for O-unit processing [9,10]. It is known that some genes participating in the synthesis of nucleotide sugar precursors that are constituents of other molecules or are involved in metabolic pathways, such as *glmU* that is responsible for the synthesis of UDP-d-GlcNAc from glucosamine-1-phosphate, are usually located elsewhere on the chromosome [11]. In *E. coli*, O-antigen synthesis can be achieved through two pathways: the Wzx/Wzy-dependent pathway and the ABC transporter-dependent pathway, each involving specific genes for O-unit processing [12].

So far, more than 180 O serogroups of *E. coli* have been identified, according to the highly variable structure of the O-antigen [13]. In addition, many putative additional serogroups were characterized by analyzing their O-AGCs, but structural data supporting their identity are missing [14,15,16]. In our previous work, an *E. coli* strain, LL004, representing a novel serogroup, was identified by using combined structural and genetic analysis [17]. Here, we established the O-antigen structure of another enteropathogenic *E. coli* strain, SD2019180, which has not been reported previously. In addition, the O-AGC of SD2019180 was characterized, and its role in O-antigen synthesis was confirmed experimentally.

## 2. Results and Discussion

### 2.1. Structural Determination of SD2019180 O-Antigen

**Figure 1:**
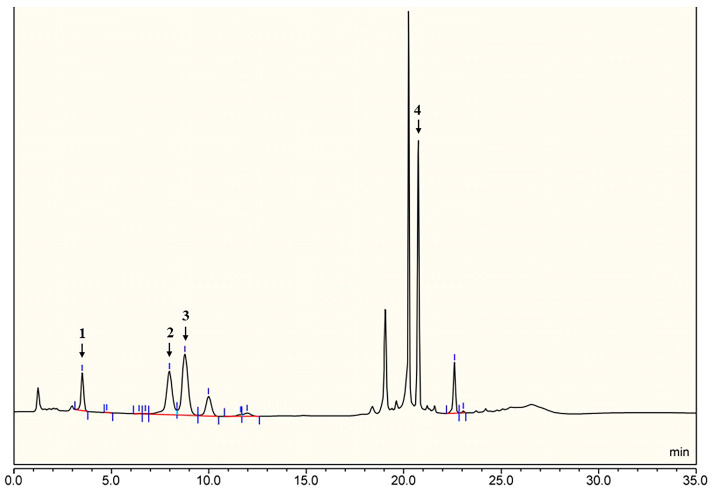
HPLC chromatogram of the hydrolyzed O-antigen from the *E. coli* strain SD2019180. The indicated peaks identify: 1, Gal*p*; 2, Fuc*p*; 3, Glc*p*N; 4, Glc*p*A.

The molecular weight of SD2019180 O-antigen was analyzed using high-performance size-exclusion chromatography (HPSEC). We observed a symmetrical and single peak, indicating that the average molecular weight of this polysaccharide is 13.5 kDa (Figure S1). Monosaccharide analysis of the fully hydrolyzed O-antigen by HPAEC-PAD revealed the presence of Gal*p*, Fuc*p*, Glc*p*N, and Glc*p*A (Figure 1). Since *N*-acetyl groups can be removed during acid-mediated full hydrolysis [18], we should consider that Glc*p*NAc could also be a constituent.

**Figure 2:**
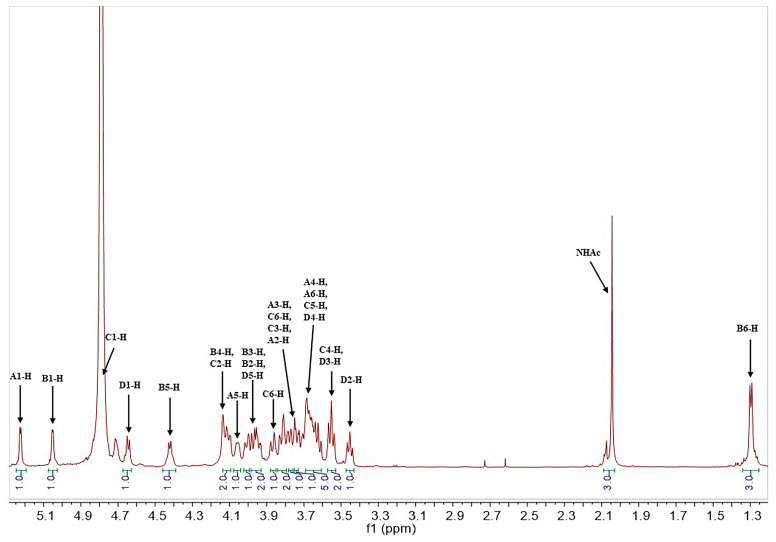
^1^H NMR spectrum of the O-specific polysaccharide from the *E. coli* strain SD2019180.

**Figure 3:**
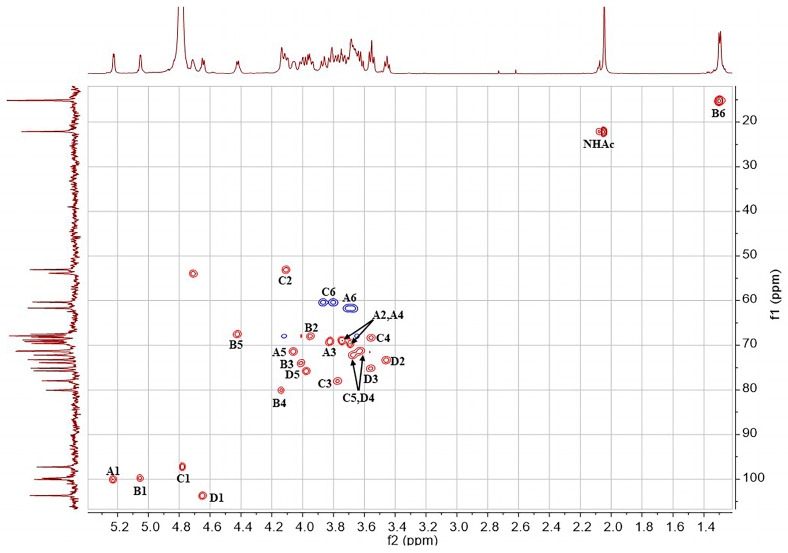
HSQC spectrum of the O-specific polysaccharide from the *E. coli* strain SD2019180.

The structure of the SD2019180 O-antigen was further analyzed using 1D (^1^H, ^13^C) and 2D (^1^H-^1^H COSY, ^13^C-edited HSQC, coupled HSQC, ^1^H-^1^H TOCSY, ^1^H-^13^C HMBC, and ^1^H-^1^H NOESY) NMR. It can be seen from the integration values of the ^1^H NMR spectrum that the SD2019180 O-antigen is a homogeneous polysaccharide (Figure 2 and Figure S2). In the upfield region (1.8–2.2 ppm) of the ^1^H NMR spectrum, one single sharp signal with an integration value of 3 can be seen, which corresponds to the methyl protons of the acetyl group (Table 1). It indicated that the repeating unit of the polysaccharide is decorated with an acetyl group. The signal at 174.5 ppm in the ^13^C NMR spectrum was assigned to carbonyl carbon (Figure S3). Another signal in the upfield region (20–23 ppm) of the ^13^C NMR spectrum was assigned to the methyl carbon of the acetyl group. Since the electron-withdrawing effect of the ester group can induce a significant downfield shift (~0.6 ppm) of the corresponding sugar ring proton signal, we concluded that there is no *O*-acetyl group in the SD2019180 O-antigen. Taking the integration value of the signal at 2.02 ppm into account, the repeating unit of this O-antigen appeared to be decorated with one *N*-acetyl group. Four signals were observed in the anomeric region (95–105 ppm) of the ^13^C-edited HSQC spectrum of the SD2019180 O-antigen (Figure 3 and Figure S4). The monosaccharides of the repeating unit of the polysaccharide are indicated by capital letters (**A**, **B**, **C**, and **D**) throughout the entire text, tables, and figures. The ^1^H NMR signal at 5.23 ppm and the ^13^C NMR signal at 100.0 ppm were assigned to the 1-H and 1-C of residue **A**, respectively (Table 1). The ^1^H NMR signal at 5.05 ppm and the ^13^C NMR signal at 99.8 ppm were assigned to the 1-H and 1-C of residue **B**, respectively. The ^1^H NMR signal at 4.78 ppm and the ^13^C NMR signal at 97.3 ppm were assigned to the 1-H and 1-C of residue **C**, respectively. The ^1^H NMR signal at 4.65 ppm and the ^13^C NMR signal at 103.7 ppm were assigned to the 1-H and 1-C of residue **D**, respectively.

**Figure 4:**
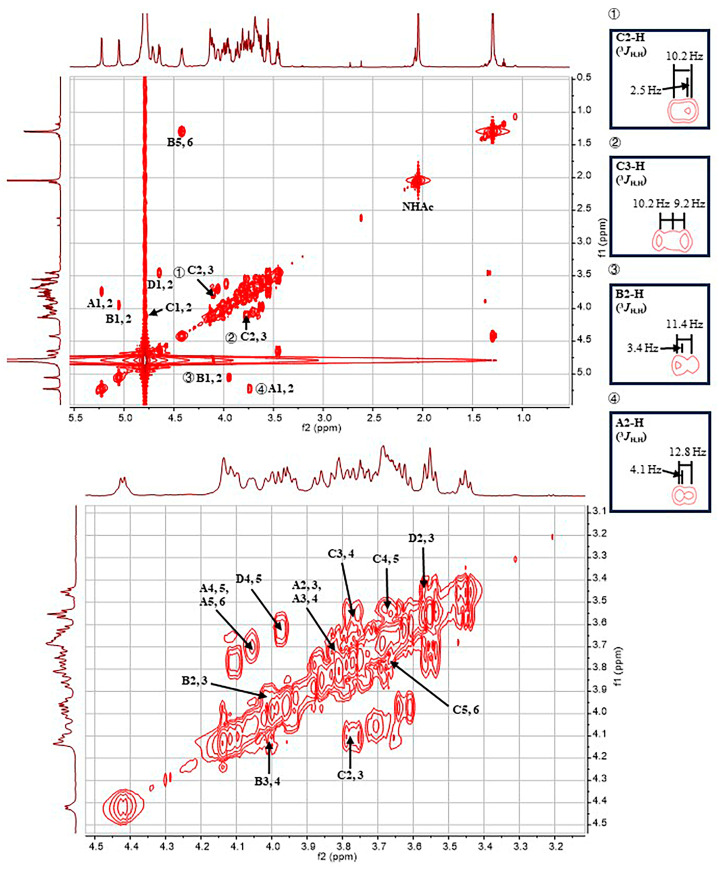
COSY spectrum of the O-specific polysaccharide from the *E. coli* strain SD2019180.

**Figure 5:**
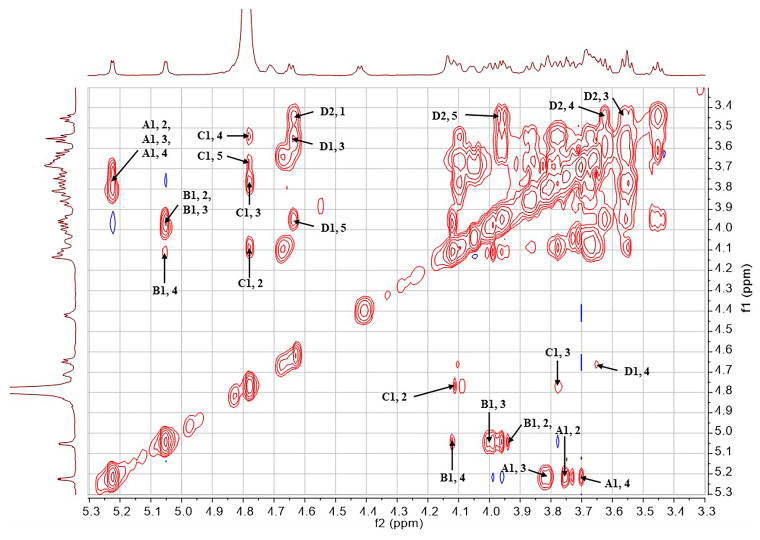
Partial TOCSY spectrum of the O-specific polysaccharide from the *E. coli* strain SD2019180.

The ^1^H NMR signals of residue **A** were assigned according to the ^1^H-^1^H COSY (Figure 4) and ^1^H-^1^H TOCSY spectra (Figure 5 and Figure S5). The 2-H and 3-H signals of residue **A** appeared at 3.74 ppm (^3^*J*_H,H_ = 12.8, 4.1 Hz) and 3.82 ppm (^3^*J*_H,H_ = 12.8 Hz), respectively. The 4-H and 5-H signals of residue **A** appeared at 3.69 ppm (singlet) and 4.06 ppm (^3^*J*_H,H_ = 6.4 Hz), respectively. The ^1^H NMR signals at 3.68 and 3.70 ppm were assigned to the 6-CH_2_ of residue **A**. The ^13^C NMR signals of the residue **A** were assigned with the aid of the signals in the HSQC spectrum. According to the coupling constants of sugar ring protons, residue **A**, with ^3^*J*_H1,H2_ of 4.1 Hz and ^1^*J*_H1,C1_ of 171.5 Hz (observed in the coupled HSQC spectrum, Figure S6), was identified as α-Gal*p*. In addition, except for **A**4-H, the ^1^H and ^13^C NMR chemical shifts of residue **A** are in agreement with NMR data reported for α-Gal*p* [18,19].

The chemical shifts of all sugar ring protons in residue **B** were assigned using the ^1^H-^1^H COSY and ^1^H-^1^H TOCSY spectra on the basis of the known chemical shift values of 1-H. The 2-H, 3-H, and 4-H signals of residue **B** appeared at 3.95 ppm (^3^*J*_H,H_ = 11.4, 3.4 Hz), 4.02 ppm (^3^*J*_H,H_ = 11.4 Hz), and 4.14 ppm (singlet), respectively. There was a signal at 4.42 ppm, which was related to the **B**4-H signal. Thus, the chemical shift of **B**5-H was determined to be 4.42 ppm (^3^*J*_H,H_ = 6.5 Hz). In addition, in the ^1^H-^13^C HMBC spectrum (Figure S7), the ^1^H NMR signal at 1.30 ppm showed a correlation with the ^13^C NMR signal at 67.5 ppm, which, in turn, was correlated with **B**5-H (4.42 ppm) in the ^13^C-edited HSQC spectrum. Therefore, the chemical shift of **B**6-H was 1.30 ppm (^3^*J*_H,H_ = 6.5 Hz), indicating that residue **B** is a 6-deoxy sugar. The ^13^C NMR signals of residue **B** were assigned with the aid of the signals in the HSQC spectrum. According to the coupling constants of sugar ring protons, residue **B**, with ^3^*J*_H1,H2_ of 3.4 Hz, and ^1^*J*_H1,C1_ of 171.9 Hz, was identified as α-Fuc*p*. In addition, the ^1^H and ^13^C NMR chemical shifts of residue **B** are in agreement with NMR data reported for α-Fuc*p* [19].

The ^1^H NMR signals of residue **C** were assigned according to the ^1^H-^1^H COSY and ^1^H-^1^H TOCSY spectra. A signal at 4.10 ppm was suspected to be related to the **C**1-H signal in the COSY spectrum. Moreover, the ^1^H NMR signal at 4.10 ppm was related to the **C**1-C signal, and the ^13^C NMR signal at 53.1 ppm was related to the **C**1-H signal in the ^1^H-^13^C HMBC spectrum. In summary, it was confirmed that the chemical shift values of **C**2-H and **C**2-C were 4.10 (^3^*J*_H,H_ = 10.2, 2.5 Hz) and 53.1 ppm, respectively. The 3-H, 4-H, and 5-H signals of residue **C** appeared at 3.77 ppm (^3^*J*_H,H_ = 10.2, 9.2 Hz), 3.55 ppm (^3^*J*_H,H_ = 9.2, 9.2 Hz), and 3.67 ppm, respectively. The ^1^H NMR signals at 3.80 and 3.87 ppm were assigned to the 6-CH_2_ of residue **C**. The ^13^C NMR signals of residue **C** were assigned with the aid of signals in the HSQC spectrum. The upfield shift of the **C**2-C signal (53.1 ppm) indicated the presence of a C-N linkage. In addition, the 2-H signal of residue **C** was found to be correlated with the carbonyl carbon (174.5 ppm) of the acetyl group in the ^1^H-^13^C HMBC spectrum, suggesting that an acetamido group is located at the 2 position of this residue. According to the coupling constants of sugar ring protons, residue **C**, with ^1^*J*_H1,C1_ of 172.1 Hz, was identified as α-Glc*p*NAc. In addition, the ^1^H and ^13^C NMR chemical shifts of residue **C** are in agreement with NMR data reported for α-Glc*p*NAc [20,21].

The chemical shifts of all sugar ring protons in residue **D** were assigned using the ^1^H-^1^H COSY and ^1^H-^1^H TOCSY spectra on the basis of the known chemical shift values of **D**1-H. The 2-H, 3-H, 4-H, and 5-H signals of residue **D** appeared at 3.45 ppm (^3^*J*_H,H_ = 8.6, 7.6 Hz), 3.57 ppm (^3^*J*_H,H_ = 9.2, 8.6 Hz), 3.63 ppm, and 3.98 ppm, respectively. The 5-H signal of residue **D** was only correlated with the **D**4-H signal in the ^1^H-^1^H COSY spectrum, indicating that residue **D** does not contain any C6 protons. In the ^1^H-^13^C HMBC spectrum, the 5-H signal of residue **D** was identified to correspond to a carbonyl carbon (169.0 ppm), indicating that residue **D** contains a C6-carboxyl group. The ^13^C NMR signals of residue **D** were assigned with the aid of signals of the HSQC spectrum. According to the coupling constants of sugar ring protons, residue **D**, with ^3^*J*_H1,H2_ of 7.6 Hz and ^1^*J*_H1,C1_ of 161.2 Hz, was identified as β-Glc*p*A. In addition, the ^1^H and ^13^C NMR chemical shifts of residue **D** are in agreement with NMR data reported for β-Glc*p*A [19]. The small ^1^H NMR signal at 2.08 ppm and the ^13^C NMR signal at 173.9 ppm were thought to be derived from residual acetic acid. An HSQC cross peak at 4.71/53.9 ppm was found to shift to 4.56/54.7 ppm in a repeated HSQC spectrum. Since there was no related cross peak in the COSY and HMBC spectra, we concluded that this signal was derived from an unknown impurity.

**Figure 6:**
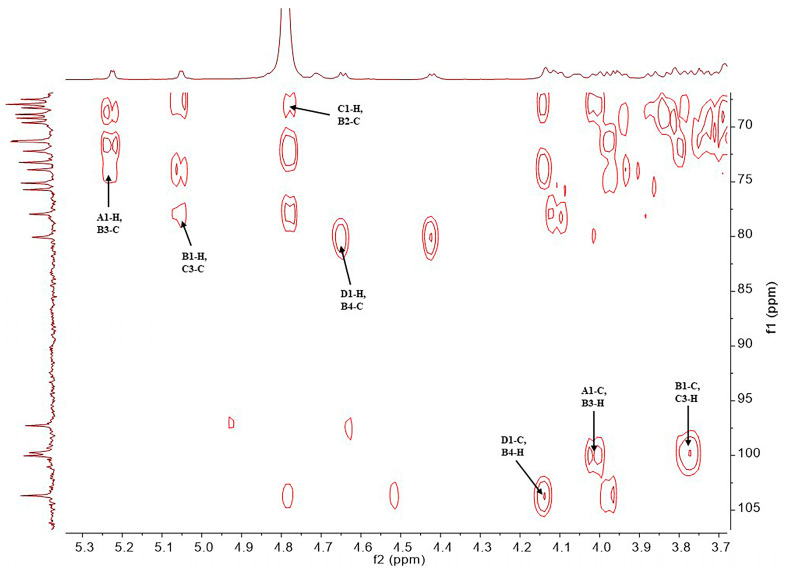
Partial HMBC spectrum of the O-specific polysaccharide from the *E. coli* strain SD2019180.

The monosaccharide composition obtained by the assignment of the NMR signals was in agreement with the results obtained from the monosaccharide analysis via HPAEC-PAD. The repeating unit of the polysaccharide was further identified through the HMBC spectrum (Figure 6). Since the **A**1-H signal (5.23 ppm) displayed HMBC correlation with the **B**3-C signal (73.9 ppm), and the **A**1-C signal (100.0 ppm) displayed HMBC correlation with the **B**3-H signal (4.02 ppm), the anomeric position of residue **A** was confirmed to be connected with the 3 position of residue **B**. Since the **B**1-H signal (5.05 ppm) displayed HMBC correlation with the **C**3-C signal (78.0 ppm), and the **B**1-C signal (99.8 ppm) displayed HMBC correlation with the **C**3-H signal (3.77 ppm), the anomeric position of residue **B** was confirmed to be linked to the 3 position of residue **C**. Since the **D**1-H signal (4.65 ppm) displayed HMBC correlation with the **B**4-C signal (80.1 ppm), and the **D**1-C signal (103.7 ppm) displayed HMBC correlation with the **B**4-H signal (4.14 ppm), the anomeric position of residue **D** was confirmed to be linked to the 4 position of residue **B**. Since the **C**1-H signal (4.78 ppm) displayed HMBC correlation with the **B**2-C signal (67.9 ppm), the anomeric position of residue **C** was confirmed to be connected with the 2 position of residue **B**.

**Figure 7:**
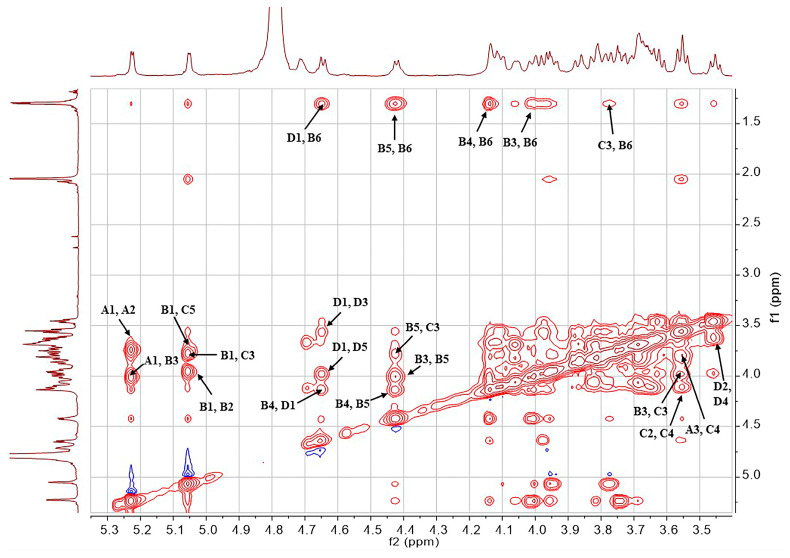
Partial NOESY spectrum of the O-specific polysaccharide from the *E. coli* strain SD2019180.

**Figure 8:**
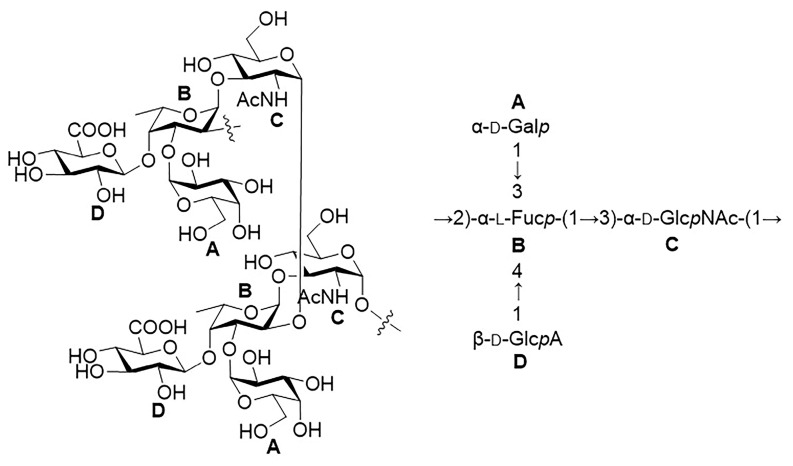
Structure of the O-specific polysaccharide from the *E. coli* strain SD2019180.

The structure of the polysaccharide was further investigated by analyzing the NOESY spectrum, particularly the NOE (nuclear Overhauser effect) signals of protons from different residues (Figure 7 and Figure S8). It was found that the **A**1-H (5.23 ppm) displayed NOE correlations with the **B**3-H (4.02 ppm). The **B**1-H (5.05 ppm) was found to be correlated to the **C**3-H (3.77 ppm) and **C**5-H (3.67 ppm). The **D**1-H (4.65 ppm) showed NOE correlations with the **B**4-H (4.14 ppm) and **B**6-H (1.30 ppm), which may come from the adjacent repeating unit. Notably, the **C**1-H (4.78 ppm) did not display NOE correlation with any protons. This information revealed that the residues **A**, **B**, and **D** are connected in a sterically crowded manner; however, the anomeric position of the residue **C** is located at the periphery of the molecule. Overall, the NOE data further confirmed the monosaccharide linkage types that are defined from the HMBC spectrum. In summary, it was confirmed that the O-antigen polysaccharide is composed of the tetrasaccharide repeating unit → 2)-[β-Glc*p*A-(1 → 4)]-[α-Gal*p*-(1 → 3)]-α-Fuc*p*-(1 → 3)-α-Glc*p*NAc-(1 → (Figure 8).

The formation of the glycosidic linkage is known to affect the ^I3^C NMR chemical shifts of both the glycone and aglycone. The effects of glycosylation depend on the configuration of the anomeric center of the glycone and on the relative absolute configuration of the glycone and aglycone. The largest differences in the ^I3^C NMR chemical shifts are observed for the carbons directly involved in the glycosidic linkage [22]. Notably, the trisaccharide fragment [β-Glc*p*A-(1 → 4)]-[α-Gal*p*-(1 → 3)]-α-Fuc*p* has also been found in the capsular polysaccharide (CPS) of *E. coli* O9:K33:H^−^. Although this CPS contains both pyruvate and *O*-acetyl groups, the NMR data of its derivative (DPS) obtained by removing the pyruvate and *O*-acetyl groups were reported [23]. Thus, a comparison between the NMR data of the *E. coli* SD2019180 O-antigen polysaccharide and the *E. coli* O9:K33:H^−^ DPS was made to investigate the absolute configuration of three monosaccharides (Table S1). It was found that the ^1^H and ^13^C NMR chemical shifts of the trisaccharide in the O-antigen are in agreement with those of the O9:K33:H^−^ DPS, particularly the 1-C of β-Glc*p*A and α-Gal*p* residues, and 1-C, 3-C, and 4-C of α-Fuc*p* residue, indicating that the three monosaccharides in the *E. coli* SD2019180 O-antigen are α-d-Gal*p*, α-l-Fuc*p*, and β-d-Glc*p*A. In addition, the disaccharide fragment α-l-Fuc*p*-(1 → 3)-α-d-Glc*p*NAc has been found in *Vibrio vulnificus* YJ016 O-antigen. In this polysaccharide, the ^I3^C NMR chemical shifts of α-l-Fuc*p* 1-C and α-d-Glc*p*NAc 3-C are 101.2 and 79.7 ppm, respectively, which are in agreement with those of the *E. coli* SD2019180 O-antigen [24]. Therefore, the α-Glc*p*NAc residue in the *E. coli* SD2019180 O-antigen was revealed to have the D-configuration. Thus, the tetrasaccharide repeating unit of the *E. coli* SD2019180 O-antigen was further confirmed as → 2)-[β-d-Glc*p*A-(1 → 4)]-[α-d-Gal*p*-(1 → 3)]-α-l-Fuc*p*-(1 → 3)-α-d-Glc*p*NAc-(1 →, which is not present in known *E. coli* serogroups.

### 2.2. The SD2019180 O-AGC Corresponds to the Identified O-Antigen Structure

**Figure 9:**
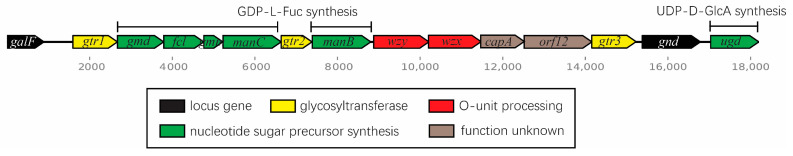
The O-antigen gene cluster of SD2019180.

The O-AGC of SD2019180 is 18,201 bp in length and consists of 13 open reading frames (*orfs*), in addition to the *galF* and *gnd* genes. Most genes are flanked by *galF* and *gnd*, while *ugd* is just downstream of *gnd*, as observed for most *E. coli* strains (Figure 9). The characteristics of the SD2019180 O-AGC *orfs* are summarized in Table 2.

The *orf3-6* and *orf8* were assigned to *gmd*, *fcl*, *gmm*, *manC*, and *manB*, respectively. The enzymes mannose-1-phosphate guanylyltransferase encoded by manC, phosphoglucomutase encoded by manB, and mannose-6-phosphate isomerase, whose coding gene *manA* is always outside the O-AGC, are involved in the synthesis of GDP-d-Man [9]. GDP-d-Man can be converted to GDP-l-Fuc, the nucleotide sugar precursor of L-Fuc, in a reaction catalyzed by the *fcl* gene product, i.e., GDP-l-fucose synthetase [25]. This is consistent with the presence of the monosaccharide residue L-Fuc in the O-antigen of SD2019180. A BLAST search showed that the product of *Orf15* is UDP-glucose 6-dehydrogenase (99% protein identity to its homologs), encoded by the *ugd* gene. Thus, we identified *orf15* as *ugd*. UDP-glucose 6-dehydrogenase was identified as the enzyme responsible for the formation of UDP-d-GlcA [26], the nucleotide sugar precursor of D-GlcA, from UDP-d-Glucose; UDP-d-GlcA is another component of the SD2019180 O-antigen. Thus, this is also consistent with the presence of D-GlcA in the SD2019180 O-antigen. The remaining two sugars of the SD2019180 O-antigen are D-GlcNAc and D-Gal. As mentioned above, the gene responsible for UDP-d-GlcNAc synthesis is outside the O-AGC, as is the *galE* gene, whose product is involved in the generation of UDP-d-Gal from UDP-d-Glucose [9]. Therefore, none of the genes participating in D-GlcNAc and D-Gal synthesis resides in the SD2019180 O-AGC.

A total of three glycosyltransferase genes in the O-AGC of SD2019180 were annotated. They are *orf2*, *orf7*, and *orf13*. Since, in most cases, the O-antigen is assembled through the Wzx/Wzy-dependent pathway, the number of glycosyltransferase genes is one less than the number of sugar residues in the O-unit, as the initial GlcNAc residue is added by WecA, whose encoding gene is within the enterobacterial common antigen cluster [27]. The α-l-Fuc-(1 → 3)-d-GlcNAc linkage in the SD2019180 O-antigen is also present; this is the only disaccharide common to a few *E. coli* serogroups, including O36, O41, O156, O159, and O168. On the other hand, these serogroups share a common glycosyltransferase gene located between the *manC* and *manB* genes, i.e., *wfeY*, as also observed for SD2019180 O-AGC. Since Orf7 and WfeY share 94% identity at the protein level, it is very reasonable that Orf7 must be responsible for the formation of the α-l-Fuc-(1 → 3)-d-GlcNAc linkage in the SD2019180 O-antigen. Another linkage revealed in the SD2019180 O-antigen, i.e., α-d-Gal-(1 → 3)-l-Fuc, is present in the O-antigen of *E. coli* O156 and is the only shared linkage between these two strains. Simultaneously, Orf2 of SD2019180 exhibited 54% identity at the protein level to the *wdbJ* gene product in O156. Thus, it is suggested that *orf2* is responsible for the transfer of D-Gal to L-Fuc, forming the α-d-Gal-(1 → 3)-l-Fuc linkage in SD2019180. Furthermore, we propose that the third glycosyltransferase gene, *orf13*, must be involved in the formation of the remaining linkage, β-d-GlcA-(1 → 4)-l-Fuc, in the SD2019180 O-antigen.

Via BLAST analysis, we found that Orf9 shares 97–100% amino acid identity with the O-antigen polymerase (Wzy), and Orf10 shares 98–100% amino acid identity with flippase (Wzx) from other *E. coli* strains. In addition, Or9 possesses 10 transmembrane domains, and Orf10 contains 13 transmembrane domains, as the typical feature of Wzy and Wzx proteins, respectively, that cooperatively assemble the O-antigen [28,29]. Therefore, *orf9* and *orf10* were identified as the genes encoding the O-antigen polymerase (*wzy*) and flippase (*wzx*), respectively, which also indicated that SD2019180 likely synthesizes its O-antigen via the Wzx/Wzy-dependent pathway. *orf11* was annotated *capA*; however, its role in O-antigen synthesis has not yet been elucidated. The function of *orf12* was not proposed using the BLAST search; therefore, *orf12* was assigned as a hypothetical protein-coding gene.

Overall, the O-AGC of SD2019180 is fully consistent with its O-antigen structure.

### 2.3. A Deletion and Complementation Test Confirmed the Role of SD2019180 O-AGC

**Figure 10:**
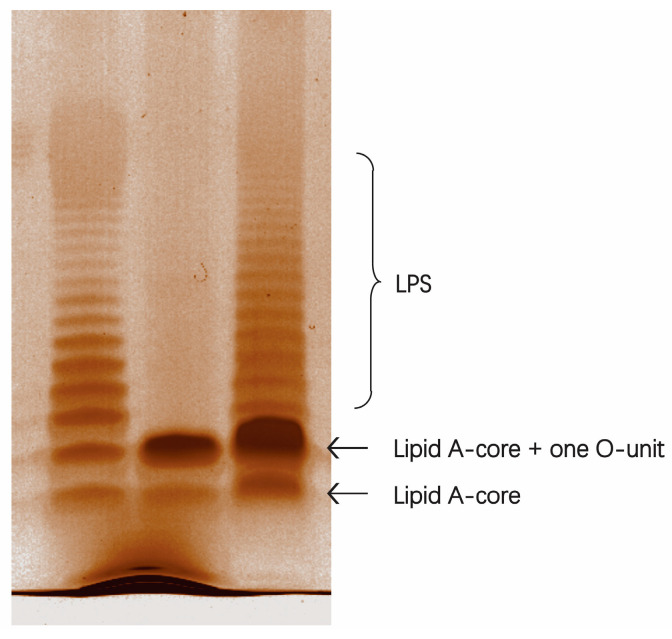
Lipopolysaccharide profiles of SD2019180 and its derivatives. The cell extracts were electrophoresed on a 12% SDS-PAGE gel and visualized via silver staining. From left to right: SD2019180, SD2019180Δ*wzy*, and SD2019180Δ*wzy*::*wzy*.

To confirm the role of SD2019180 O-AGC in O-antigen synthesis, a deletion and complementation test was performed. As shown in the LPS profile (Figure 10), the SD2019180 wild-type strain showed a completely smooth LPS phenotype, with a lipid A-core band plus serial bands of O-units of different molecular weights. On the contrary, the *wzy*-deleted strain, SD2019180Δ*wzy*, showed only a semi-rough LPS profile with a single O-unit substitution on the lipid A-core. Moreover, the complete LPS phenotype could be restored in the *wzy*-complemented strain, SD2019180Δ*wzy*::*wzy*. Collectively, these results indicated that the O-AGC we characterized is involved in the synthesis of the SD2019180 O-antigen and that the assembly of the SD2019180 O-antigen is mediated by the Wzx/Wzy-dependent pathway.

## 3. Materials and Methods

### 3.1. LPS and O-Specific Polysaccharide Extraction

The bacteria were grown in LB medium pH 7.0, under constant aeration at 37 °C to late log phase. The bacterial cells were collected, washed, and dried [30]. The collected cells were broken by the freeze-drying method, then dissolved in a 90% water–phenol solution and shaken at 120 rpm on a shaking table at 65 °C for 30 min [31]. After centrifugation at the low speed of 4000 rpm at 4 °C for 30 min, we collected the upper water phase and dialyzed it in distilled water until it was free from phenol. After freeze drying, the crude product LPS was dissolved in distilled water and treated with deoxyribonuclease, ribonuclease, and protease K in sequence. The supernatant was collected via centrifugation at 8000 rpm at 4 °C for 30 min, and water-saturated phenol was added, mixing well. A centrifugation at 4000 rpm for 30 min was performed at 4 °C before dialysis and freeze drying to obtain LPS. Then, the extracted LPS was treated with 2 mg/mL and 2% (*v*/*v*) acetic acid at 100 °C for 3 h, and the precipitated lipid A was removed via ultracentrifugation (13,000× *g*, 30 min, 4 °C). After purification on a Sephadex G-50 column (J&K Scientific, Shanghai, China) with 0.05 M pyridine acetate buffer (pH 4.5), the O-specific polysaccharide was obtained.

High-efficiency particle size-exclusion chromatography (HPSEC) was used to determine the molecular weight of the O-specific polysaccharide [31]. The polysaccharide was analyzed via Waters 1525 high-performance liquid chromatography (HPLC) with an Ultrahydrogel Linear (7.8 mm × 30.0 cm) column (Waters Corp., Milford, MA, USA). The mobile phase was 0.1 mol/L NaNO_3_, and the flow rate was 0.5 mL/min. The eluent was monitored with a Waters 2410 refractive index detector (Waters Corp., Milford, MA, USA). The column temperature was kept at 40 °C. A polysaccharide solution at the concentration of 5 mg/mL in water was used as the test solution. The injection volume was 50 μL. Calibration standards were plotted with five glucan standards (Mw 2.70, 9.75, 135.03, 300.60, and 2000 kDa) and glucose (Mw 180 Da).

### 3.2. Monosaccharide Analysis

The O-specific polysaccharide was hydrolyzed with 2 M trifluoroacetic acid (120 °C, 2 h) at 1.5 mg/mL, and the excess acid was removed by the addition of methanol and evaporation [24]. The monosaccharides were analyzed via high-performance anion-exchange chromatography-pulsed amperometric detection (HPAEC-PAD) [32] using the ICS-5000+ ion chromatography system, which consists of a four-element pump, a temperature-controlled column manager, and an ED5000 PAD electrochemical cell. The ED5000 PAD electrochemical cell consists of an Au working electrode and a pH-Ag/AgCl reference electrode (Thermo Fisher Scientific, Waltham, MA, USA). A Dionex CarboPac PA20 anion-exchange column was used, consisting of an analytical column (3 × 150 mm) and a protective column (3 × 50 mm). The column temperature was 30 °C. The injection volume was 25 μL. A 5 mmol/L sodium hydroxide (NaOH) solution (solvent A) and a 5 mmol/L sodium acetate (NaOAc) solution (solvent B) containing 250 mmol/L were used as the mobile phase. The gradient elution conditions were 100% A (0–15 min), 80–0% A, 20–100% B (15–24 min), and 100% A (24–35 min). The flow rate was 0.5 mL/min. According to the retention time of the standard monosaccharides, the types of monosaccharides in the O-specific polysaccharide were identified. Ten monosaccharides, including D-Gal, D-Glc, D-Man, L-Fuc, L-Rha, D-GalN, D-GlcN, D-Fru, D-GalA, and D-GlcA, were used as standards.

### 3.3. NMR Analysis

The NMR spectroscopic samples were freeze-dried with 99.9% D_2_O in exchange for deuterium and then tested as a solution of 99.95% D_2_O. The NMR spectra were recorded with the Bruker Ascend 600 MHz spectrometer (Bruker, Bremen, Germany). The chemical shifts of the ^1^H NMR were referenced to the solvent residual peak (*δ*_H_ 4.79 ppm) [33]. The Bruker software TopSpin 4.1.0 was used to collect and process the NMR data. For the ^1^H NMR experiment, the pulse program was zg30, the acquisition time was 2.75 s, the temperature was 27 °C, and the number of scans was 16. For the ^13^C NMR experiment, the pulse program was zgpg30, the acquisition time was 0.92 s, the temperature was 27 °C, and the number of scans was 15,360. For the ^1^H-^1^H COSY experiment, the pulse program was cosygpprqf, the acquisition time was 0.24 s, the temperature was 27 °C, and the number of scans was 16. For the ^13^C-edited HSQC experiment, the pulse program was hsqcedetgpsisp2.3, the acquisition time was 0.13 s, the temperature was 27 °C, and the number of scans was 32. For the ^1^H-^13^C HMBC experiment, the pulse program was hmbcgpndqf, the acquisition time was 0.16 s, the temperature was 27 °C, and the number of scans was 128. For the coupled HSQC experiment, the pulse program was hsqcedetgpsisp2.3, the acquisition time was 0.13 s, the temperature was 27 °C, and the number of scans was 32. For the ^1^H-^1^H TOCSY experiment, the pulse program was mlevphpr.2, the acquisition time was 0.17 s, the temperature was 40 °C, and the number of scans was 16. For the ^1^H-^1^H NOESY experiment, the pulse program was noesygpphpr, the acquisition time was 0.17 s, the temperature was 27 °C, and the number of scans was 32.

### 3.4. Bacterial Strains, Plasmids, and Growth Conditions

The bacterial strains, plasmids, and primers used in this study are listed in Table S2. SD2019180 was agglutinated against the antisera targeting almost all serogroups of *E. coli* (IM-EH001, Tianjin Biochip Co., Ltd., Tianjin, China) and was tested by using SerotypeFinder, a publicly available tool for the whole-genome-based in silico serotyping of *E. coli*. However, no positive result was obtained, suggesting SD2019180 is a putative novel serogroup. To knock out *wzy* in SD2019180, a two-step homologous recombination with the pRE112 plasmid containing the *sacB* counter-selectable marker was performed as described previously [34]. For the complementation test, the *wzy* gene with optimized Shine–Dalgarno sequence was cloned into pBAD33 under the control of the P_BAD_ promoter; then the resultant plasmid was introduced into SD2019180Δ*wzy* via electroporation, generating the complementary strain SD2019180Δ*wzy*:: *wzy*. All strains used for sequencing and gene manipulation were cultured in Luria–Bertani (LB) medium at 37 °C. When necessary, the cultures were supplemented with chloramphenicol (25 μg/mL). To induce *wzy* expression under the control of pBAD33, L-arabinose (0.5 mg/mL) was added to mid-log phase cultures, and the cultures were incubated for an additional hour.

### 3.5. Genome Sequencing and Annotation

The genomic DNA of SD2019180 was extracted from 5 mL of an overnight bacterial culture using a DNA extraction kit (Tiangen, Beijing, China). Then, the genomic DNA was sheared, polished, and prepared using the Illumina Sample Preparation Kit. Genomic libraries containing 500 bp paired-end inserts were constructed, and sequencing was then performed with the Solexa sequencing technologies (Illumina Inc., San Diego, CA, USA) to obtain approximately a 100-fold coverage. The obtained reads were assembled using the de novo genome assembly program Velvet to generate a multi-contig draft genome. Next, Artemis was used to annotate the genes [35]. BLAST and PSI-BLAST [36] were used to search genes and proteins against in databases, including GenBank (www.ncbi.nlm.nih.gov/genbank (accessed on 15 September 2020)) and Pfam protein motif databases (pfam.sanger.ac.uk (accessed on 15 September 2020)). TMHMM v2.0 (http://www.cbs.dtu.dk/services/TMHMM-2.0/ (accessed on 15 September 2020)) was used to identify potential transmembrane domains within the protein sequences. The O-AGC sequence of SD2019180 was deposited in GenBank under accession number OR256802.

### 3.6. SDS-PAGE Analysis of LPS

The bacterial strains were grown overnight, and then the cultures were inoculated into LB broth and grown at 37 °C to the mid-log phase at a final optical density OD600 = 0.8. Then, LPS for sodium dodecyl sulphate polyacrylamide gel electrophoresis (SDS-PAGE) analysis was prepared using the hot aqueous–phenol method, as previously described [37]. The extracted LPS was separated using a 12% SDS-PAGE gel at 50 V for 30 min and 100 V for 2 h and, subsequently, was visualized via silver staining using the Fast Silver Stain Kit (no. P0017S, Beyotime, Shanghai, China). The gel image was captured using a GS900 Calibrated Densitometer (BioRad Laboratories, Hercules, CA, USA).

## 4. Conclusions

In this work, we determined the structure of the O-antigen from an *E. coli* strain, SD2019180. We found that it is composed of a tetrasaccharide biological repeating unit, → 2)-[β-d-Glc*p*A-(1 → 4)]-[α-d-Gal*p*-(1 → 3)]-α-l-Fuc*p*-(1 → 3)-α-d-Glc*p*NAc-(1 →. The structure of this polysaccharide is totally different from those of all the known *E. coli* O-antigens. We also defined the O-AGC via genomic analysis and confirmed its role in O-antigen synthesis experimentally. Considering the negative results in the immune agglutination reaction and in the in silico serotyping test in comparison with all known *E. coli* serogroups, the data presented in this work strongly indicate that SD2019180 is a novel serogroup of *E. coli*.

## Figures and Tables

**Table 1:** ^1^H and ^13^C NMR chemical shifts of the O-specific polysaccharide from the *E. coli* strain SD2019180.

Residue	Chemical Shifts (ppm)						
	1-H/1-C	2-H/2-C	3-H/3-C	4-H/4-C	5-H/5-C	6-H/6-C	NAc (C=O)
**A** α-d-Gal*p* ^1^*J*_H1,C1_ 171.5 Hz	5.23 (^3^*J*_H,H_ 4.1 Hz)/100.0	3.74 (^3^*J*_H,H_ 12.8, 4.1 Hz)/68.9	3.82 (^3^*J*_H,H_ 12.8 Hz)/69.2	3.69 (singlet)/69.6	4.06 (^3^*J*_H,H_ 6.4 Hz)/71.3	3.68, 3.70/61.7	-
**B** α-l-Fuc*p* ^1^*J*_H1,C1_ 171.9 Hz	5.05 (^3^*J*_H,H_ 3.4 Hz)/99.8	3.95 (^3^*J*_H,H_ 11.4, 3.4 Hz)/67.9	4.02 (^3^*J*_H,H_ 11.4 Hz)/73.9	4.14 (singlet)/80.1	4.42 (^3^*J*_H,H_ 6.5 Hz)/67.5	1.30 (^3^*J*_H,H_ 6.5 Hz)/15.2	-
**C** α-d-Glc*p*NAc ^1^*J*_H1,C1_ 172.1 Hz	4.78/97.3	4.10 (^3^*J*_H,H_ 10.2, 2.5 Hz)/53.1	3.77 (^3^*J*_H,H_ 10.2, 9.2 Hz)/78.0	3.55 (^3^*J*_H,H_ 9.2, 9.2 Hz)/68.3	3.67/72.2	3.80, 3.87/60.4	2.04/22.2 (174.5)
**D** β-d-Glc*p*A ^1^*J*_H1,C1_ 161.2 Hz	4.65 (^3^*J*_H,H_ 7.6 Hz)/103.7	3.45 (^3^*J*_H,H_ 8.6, 7.6 Hz)/73.3	3.57 (^3^*J*_H,H_ 9.2, 8.6 Hz)/75.2	3.63/71.3	3.98/75.8	-/169.0	-

**Table 2:** Characteristics of the *orfs* in SD2019180 O-AGC.

orf No.	Gene Name	Position of Gene	G+C Content (%)	Similar Protein(s), Strain(s) (Genbank Accession No.)	%Identical/%Similar (Total No. of aa)	Putative Function of the Protein
1	*galF*	1.894	51.78	GalU regulator GalF, [*Escherichia coli*] (EEW6173515.1)	100/100 (297)	GalU regulator GalF
2	*gtr1*	1594.2673	31.01	Glycosyltransferase family 4 protein, [*Escherichia coli*] (EII8714459.1)	54/74 (359)	Glycosyltransferase
3	*gmd*	2674.3792	42.6	GDP-mannose 4,6-dehydratase, [*Escherichia coli*] (WP_096321002.1)	99/100 (372)	GDP-mannose 4,6-dehydratase
4	*fcl*	3796.4761	39.23	GDP-l-fucose synthase, [*Escherichia coli*] (WP_044695018.1)	99/100 (321)	GDP-l-fucose synthase
5	*gmm*	4764.5225	37.22	GDP-mannose mannosyl hydrolase, [*Shigella sonnei*] (EGD4982807.1)	99/100 (153)	GDP-mannose mannosyl hydrolase
6	*manC*	5231.6637	41.43	mannose-1-phosphate guanylyltransferase/mannose-6-phosphate isomerase, [*Shigella sonnei*] (EGD4982806.1)	99/99 (468)	mannose-1-phosphate guanylyltransferase/mannose-6-phosphate isomerase
7	*gtr2*	6637.7383	34.27	Glycosyltransferase, [*Escherichia coli*] (MCB6245792.1)	99/99 (248)	Glycosyltransferase
8	*manB*	7389.8807	36.01	Phosphomannomutase, [*Escherichia coli*] (WP_054486156.1)	100/100 (472)	Phosphomannomutase
9	*wzy*	8876.10213	32.21	O-antigen polysaccharide polymerase Wzy, [*Escherichia coli*] (HCK1104133.1)	90/98 (445)	O-antigen polymerase
10	*wzx*	10,203.11477	30.03	Oligosaccharide flippase family protein, [*Escherichia coli*] (EFC0723458.1)	98/99 (424)	Flippase
11	*capA*	11,467.12522	30.96	CapA family protein, [*Escherichia coli*] (WP_205849603.1)	99/99 (351)	CapA
12	*orf12*	12,519.14159	29.06	Hypothetical protein, [*Escherichia coli*] (EIP2350151.1)	99/100 (546)	Hypothetical protein
13	*gtr3*	14,156.15229	31.28	Glycosyltransferase family 4 protein, [*Escherichia coli*] (EFN6163013.1)	99/99 (357)	Glycosyltransferase
14	*gnd*	15,380.16786	50.31	NADP-dependent phosphogluconate dehydrogenase, [*Escherichia coli*] (WP_251885442.1)	100/100 (468)	Phosphogluconate dehydrogenase
15	*ugd*	17,035.18201	43.87	UDP-glucose 6-dehydrogenase, [*Escherichia coli*] (WP_042048836.1)	99/100 (338)	UDP-glucose 6-dehydrogenase

## Data Availability

Genbank accession number OR256802.

## Supplementary Materials

The following supporting information can be downloaded at: https://www.mdpi.com/article/10.3390/ijms242015040/s1.
