# Supplementary material for: Structural and Genetic Identification of the O-Antigen from an Escherichia coli Isolate, SD2019180, Representing a Novel Serogroup
Source: Int J Mol Sci. 2023 Oct 10;24(20):15040. doi: 10.3390/ijms242015040 (PMC10606467; doi:10.3390/ijms242015040)
Supplement: Supplementary file 1 [file ijms-24-15040-s001.zip › Table S1.pdf]

Table S1. Comparison of ^{1}H and ^{13}C NMR chemical shifts between the O-specific polysaccharide from the *E. coli* strain SD2019180 and the DPS of *E. coli* O9:K33:H-.

Residue		Chemical shifts (ppm)					
		**1-H/1-C**	**2-H/2-C**	**3-H/3-C**	**4-H/4-C**	**5-H/5-C**	**6-H/6-C**
α-D-Gal*p*	SD2019180 O-antigen	5.23/100.0	3.74/68.9	3.82/69.2	3.69/69.6	4.06/71.3	3.68, 3.70/61.7
	O9:K33:H- DPS	5.26/101.3	3.78/69.6	3.91/70.2	4.01/70.6	4.15/72.2	3.72, 3.72/62.6
α-L-Fuc*p*	SD2019180 O-antigen	5.05/99.8	3.95/67.9	4.02/73.9	4.14/80.1	4.42/67.5	1.30/15.2
	O9:K33:H- DPS	5.29/100.2	4.05/69.4	4.05/75.6	4.11/81.2	4.41/68.4	1.31/16.2
β-D-Glc*p*A	SD2019180 O-antigen	4.65/103.7	3.45/73.3	3.57/75.2	3.63/71.3	3.98/75.8	-/169.0
	O9:K33:H- DPS	4.55/104.5	3.46/75.5	3.79/76.9	3.88/78.7	3.76/78.4	-/174.9
