# Supplementary material for: Structural and Genetic Identification of the O-Antigen from an Escherichia coli Isolate, SD2019180, Representing a Novel Serogroup
Source: Int J Mol Sci. 2023 Oct 10;24(20):15040. doi: 10.3390/ijms242015040 (PMC10606467; doi:10.3390/ijms242015040)
Supplement: Supplementary file 1 [file ijms-24-15040-s001.zip › Table S2.pdf]

Table S2. Strains, plasmids, and primers used in this study.

Strain/plasmid/primer	Description
Bacterial strain	
EPEC025	Wild-type strain
EPEC025Δ*wzy*	Deletion of EPEC025 *wzy* gene
EPEC025Δ*wzy*::*wzy*	EPEC025Δ*wzy* complemented with pBD33-wzy
Plasmid	
pRE112	Allelic exchange vector with *sacB*, chloramphenicol resistant
pRE112-updown	pRE112 fused with EPEC025 *wzy* up- and down- homologous sequences
pBD33	Expression vector with chloramphenicol selection
pBD33-wzy	pBD33 inserted with EPEC025 *wzy* plus Shine-Dalgarno box
Primer	Nucleotide sequences (5'-3') [a]
112F	CATCGTATTTCTGGTGCGTACCCTGATAAATGCTTCAATAATGG, forward primer for pRE112 linearization
112R	ATTGCGGCTAAAGCGTTAGTCAACAGCTCATTTCAGAATGG, reverse primer for pRE112 linearization
upF	CCATTATTGAAGCATTTATCAGGGTACGCACCAGAAATACGATG, forward primer for *wzy* upload sequence
upR	AACTCTTGATACCAGCGGAGGTATCGCATAGCCAACA, reverse primer for *wzy* upload sequence
downF	TGTTGGCTATGCGATACCTCCGCTGGTATCAAGAGTT, forward primer for *wzy* download sequence
downR	CCATTCTGAAATGAGCTGTTGACTAACGCTTTAGCCGCAAT, reverse primer for *wzy* download sequence
wzycF	CGG*GGTACC***AGGAGG**AAAAATGAAAAGAAATTTTTATATACTGT, forward primer for *wzy* cloning, digested with KnpI
wzycR	CCC*AAGCTT*TTAATTGGCAAGATTAATCCT, reverse primer for *wzy* cloning, digested with HindIII

[a] Boldface characters indicate Shine-Dalgarno box and restriction sites are italic.
