# Supplementary figures and images for: Structural and Genetic Identification of the O-Antigen from an Escherichia coli Isolate, SD2019180, Representing a Novel Serogroup
Source: Int J Mol Sci. 2023 Oct 10;24(20):15040. doi: 10.3390/ijms242015040 (PMC10606467; doi:10.3390/ijms242015040)

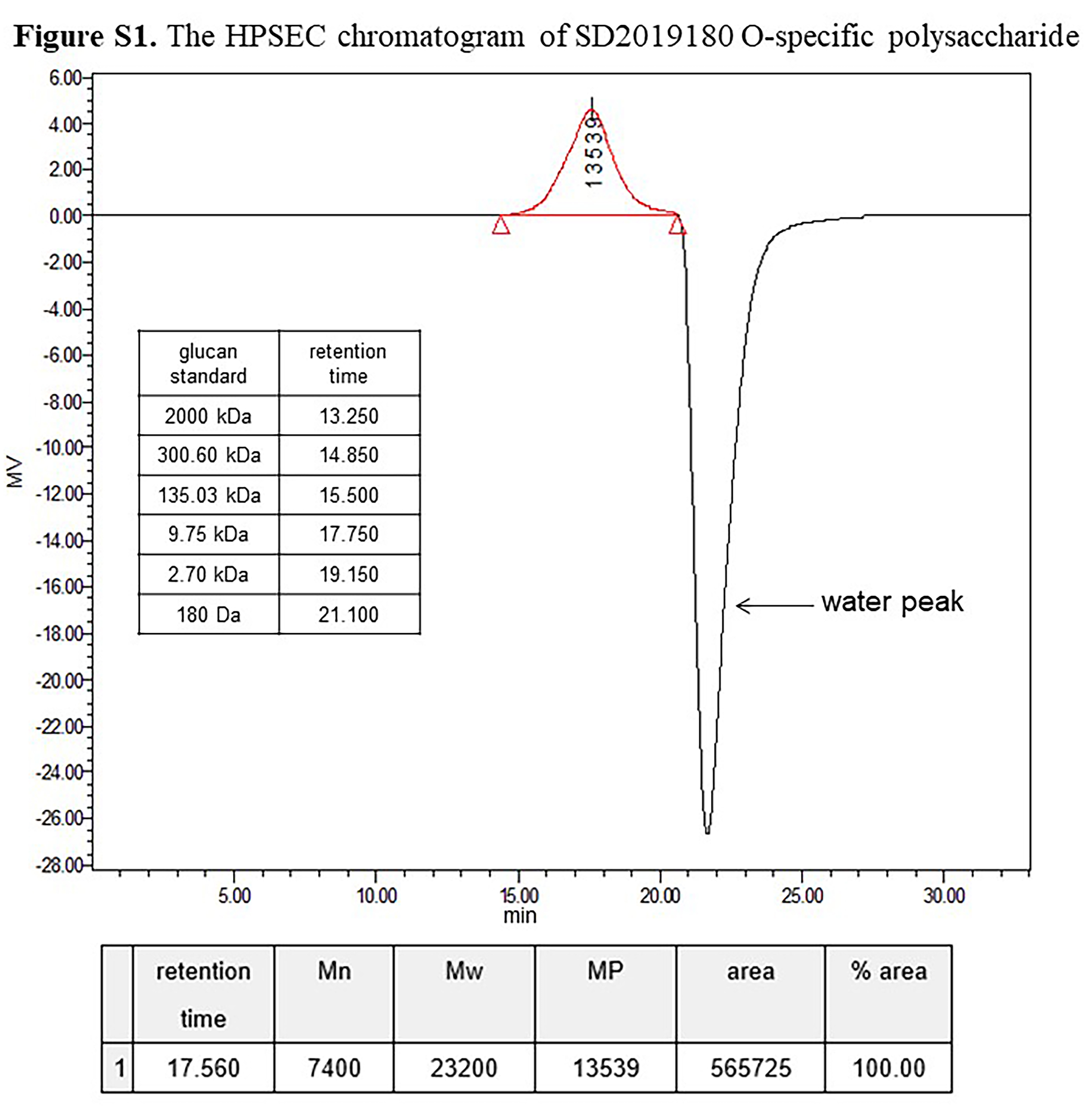

Supplement: Supplementary file 1 [file ijms-24-15040-s001.zip › Figure S1.jpg]

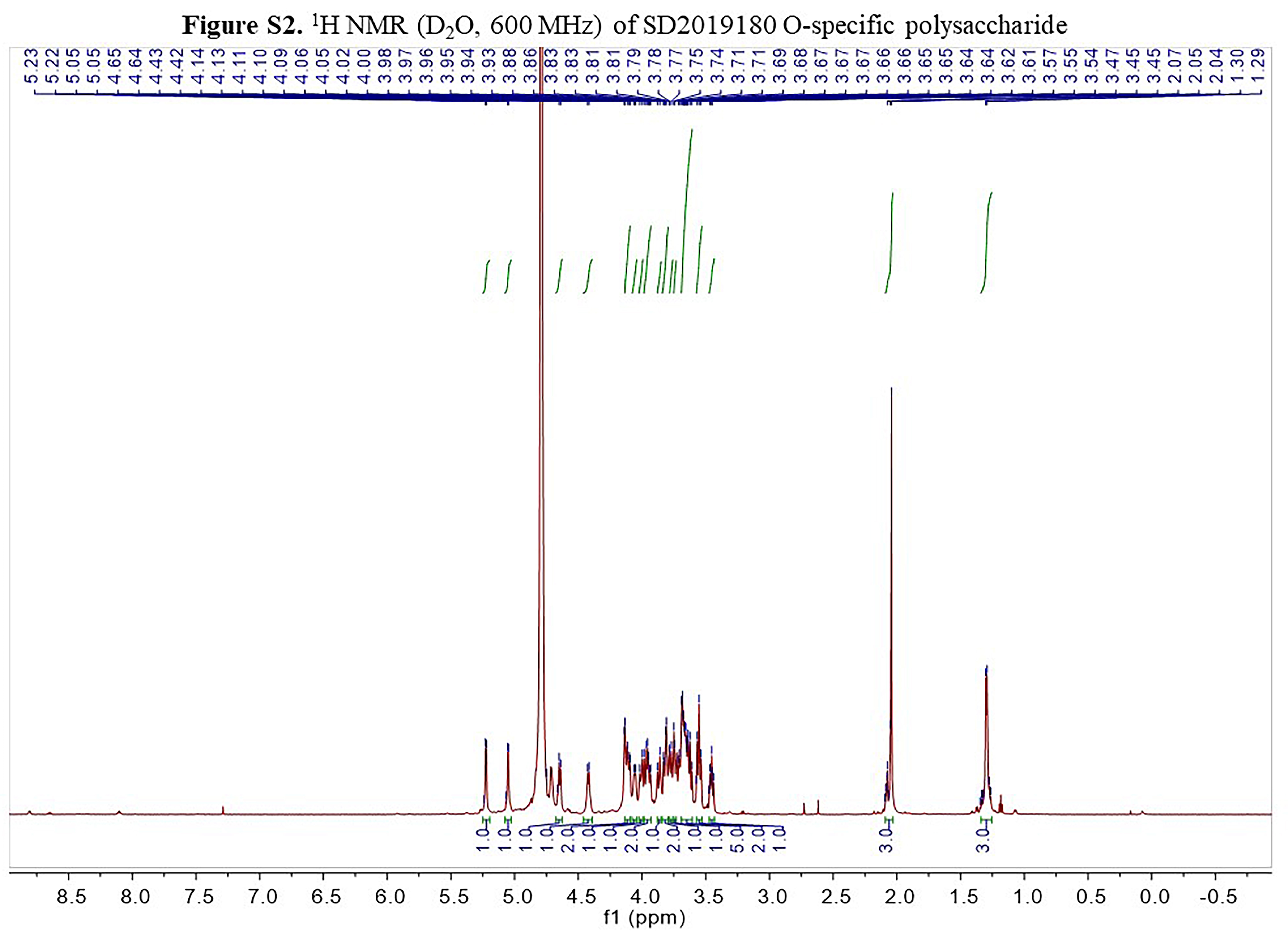

Supplement: Supplementary file 1 [file ijms-24-15040-s001.zip › Figure S2.jpg]

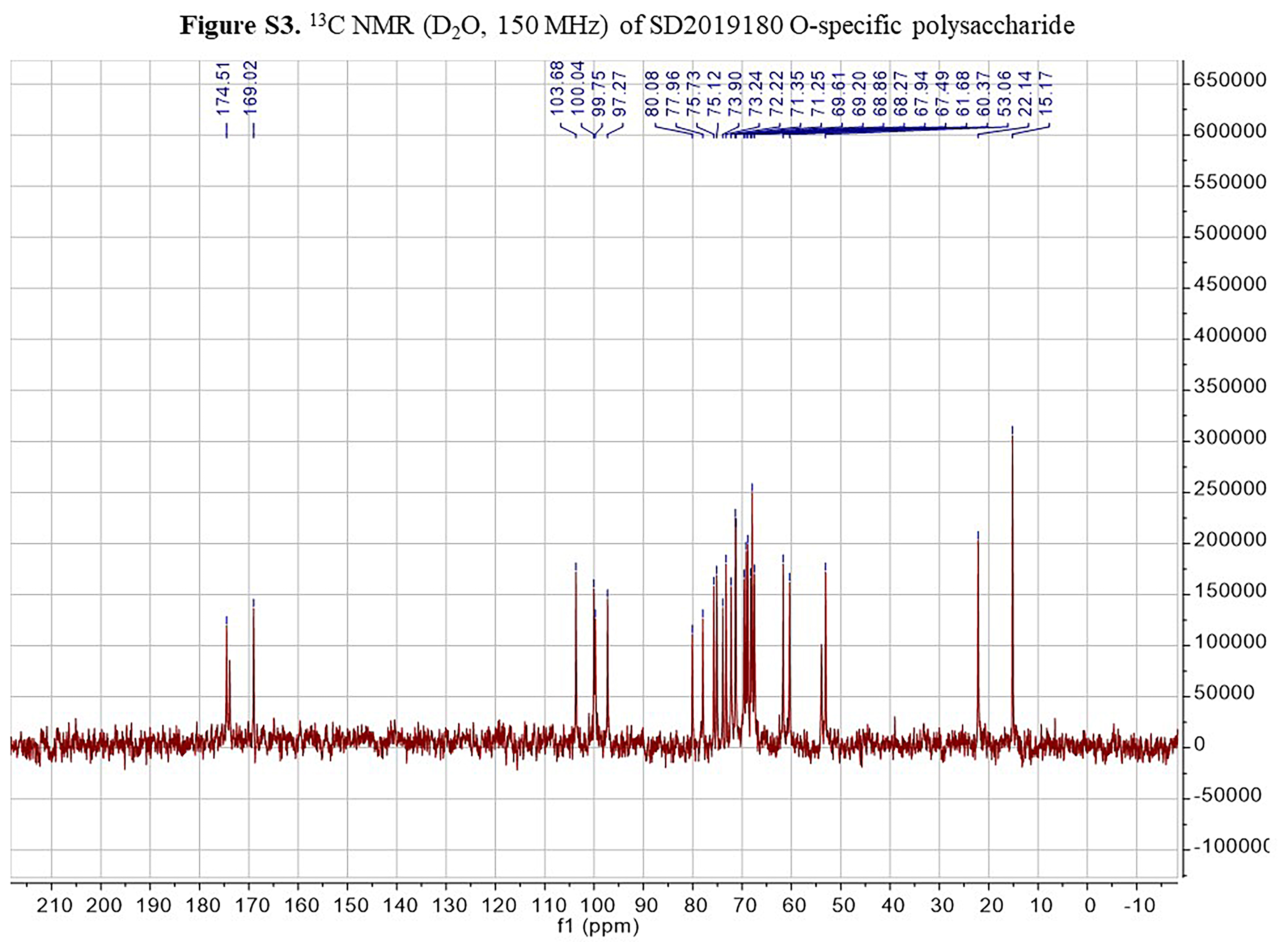

Supplement: Supplementary file 1 [file ijms-24-15040-s001.zip › Figure S3.jpg]

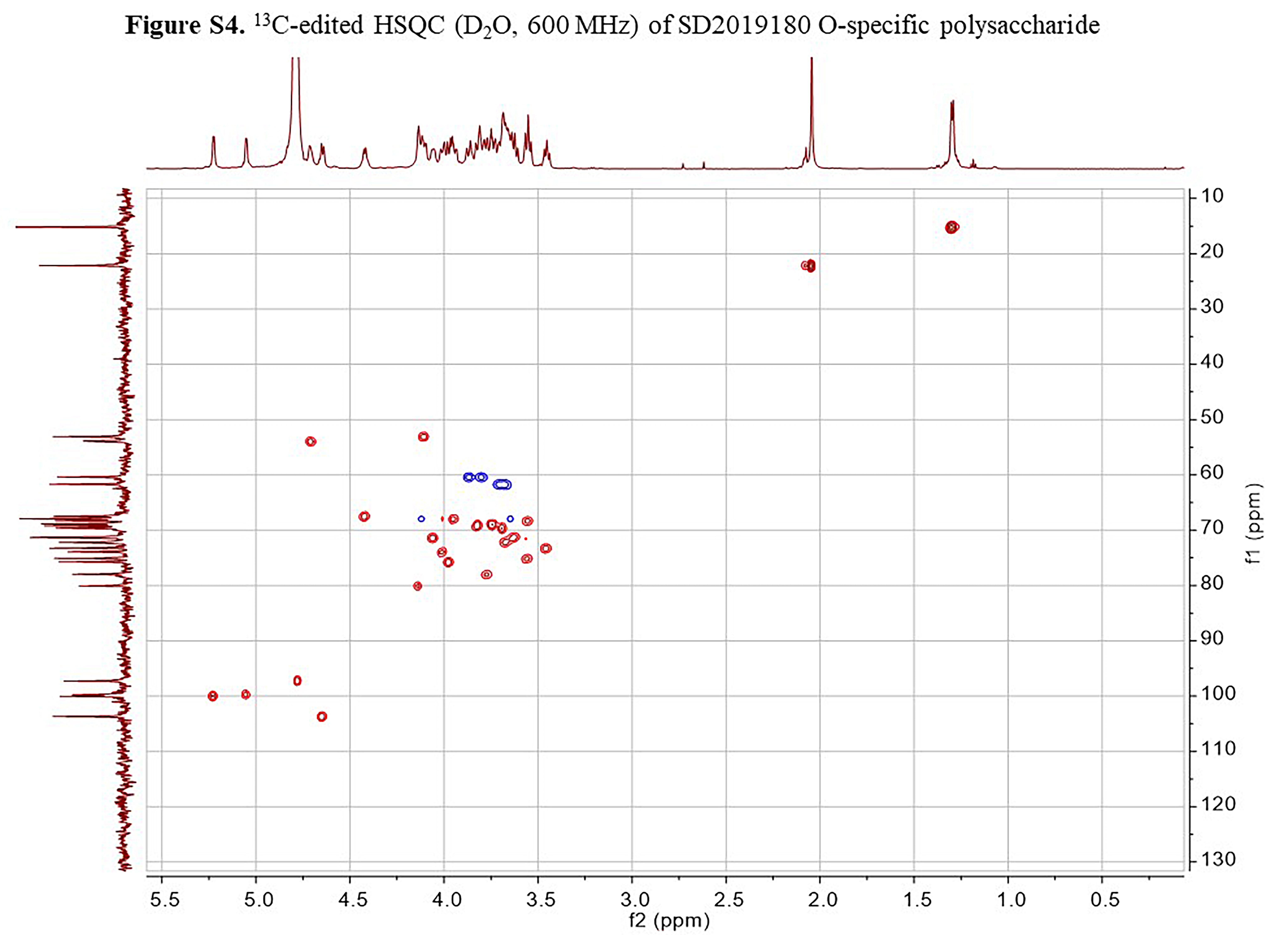

Supplement: Supplementary file 1 [file ijms-24-15040-s001.zip › Figure S4.jpg]

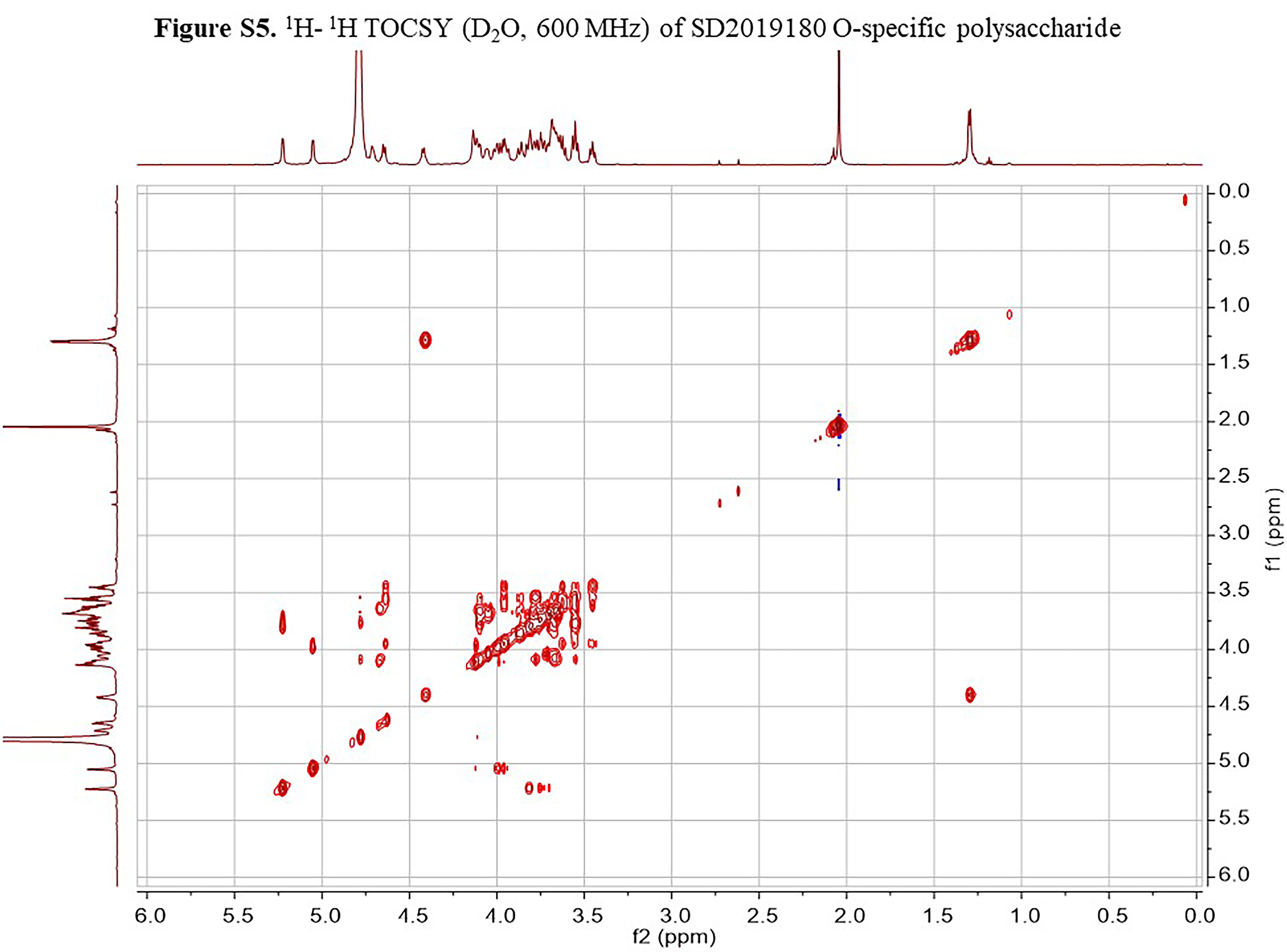

Supplement: Supplementary file 1 [file ijms-24-15040-s001.zip › Figure S5.jpg]

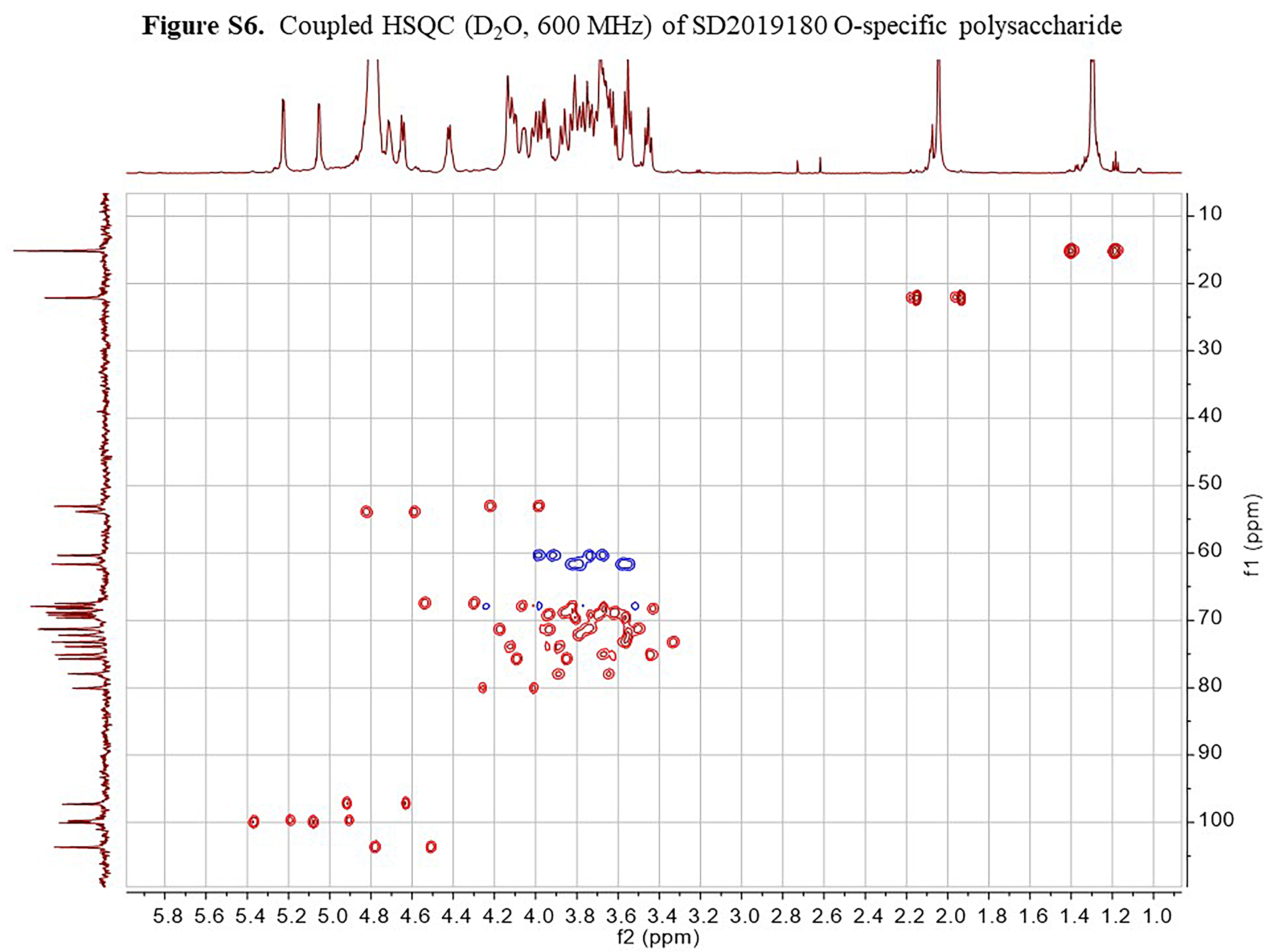

Supplement: Supplementary file 1 [file ijms-24-15040-s001.zip › Figure S6.jpg]

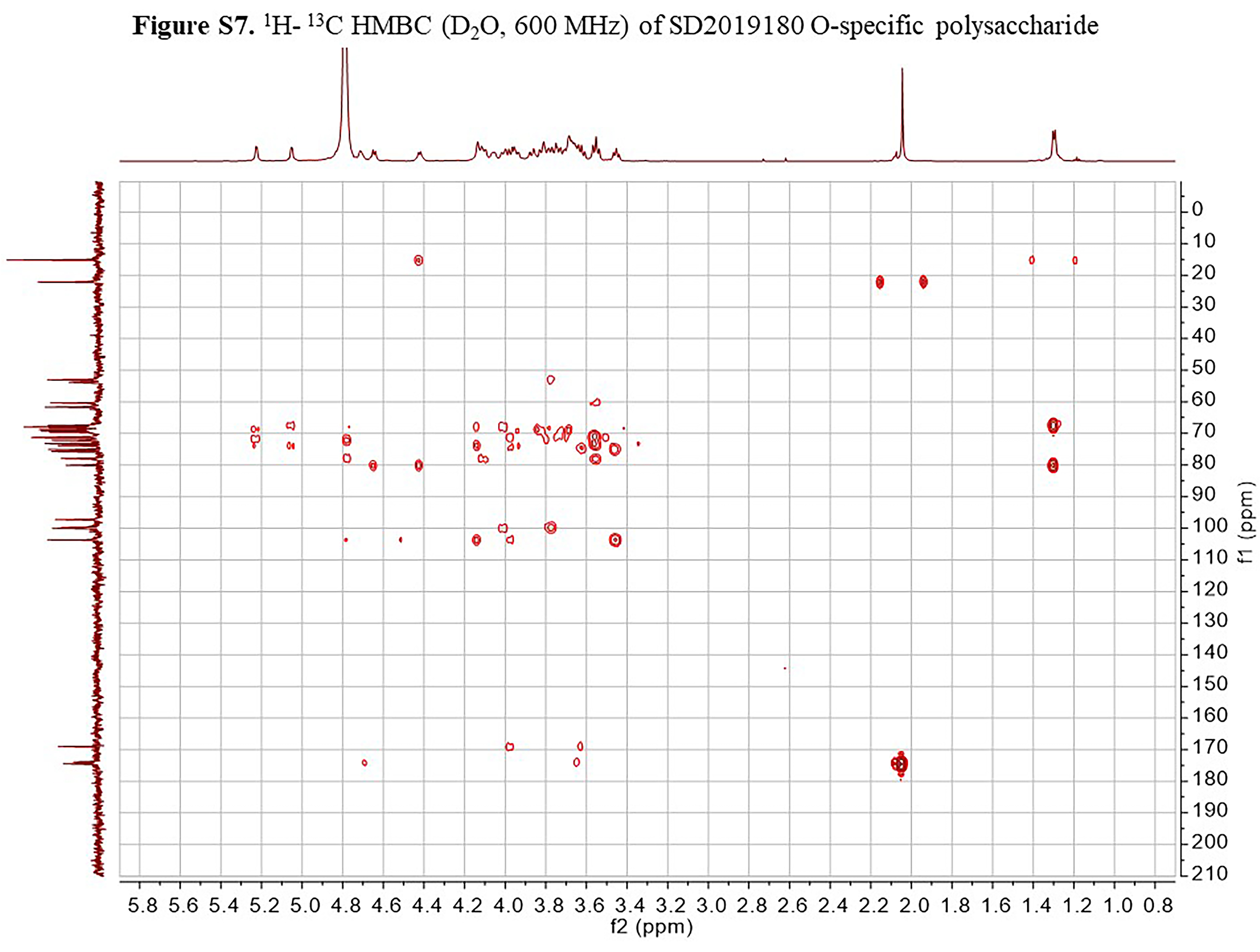

Supplement: Supplementary file 1 [file ijms-24-15040-s001.zip › Figure S7.jpg]

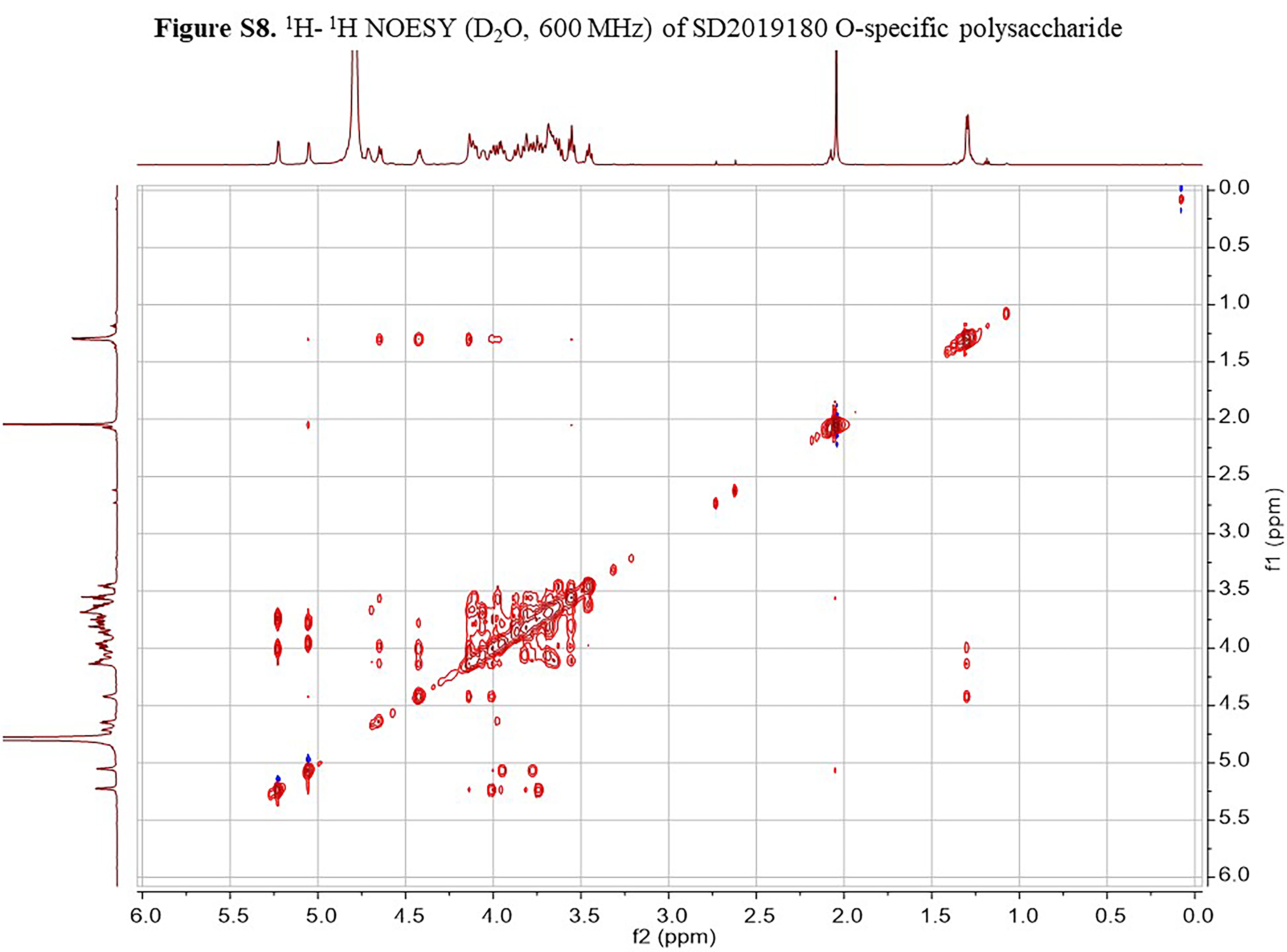

Supplement: Supplementary file 1 [file ijms-24-15040-s001.zip › Figure S8.jpg]
